# Twelve-month real-world outcomes of sparsentan in a multicenter Spanish IgA nephropathy cohort

**DOI:** 10.1093/ckj/sfag298

**Published:** 2026-09-01

**Authors:** Clara García-Carro, Antolina Rodríguez-Moreno, Lucía Cordero, María Vanessa Pérez-Gómez, Helena Marco Rusiñol, Enrique Pasache, Alejandro Morales, Rocío Zamora, Diego Parra, Juan Villa, Elena Guillén-Olmos, Verónica Coll-Brito, Álvaro Álvarez, Juliana Bordignon, Carola Arcal, Olga Gracia, Patricia Delgado, María José Soler, Ana I Sánchez-Fructuoso

**Affiliations:** Nephrology Department, San Carlos Clinical University Hospital, National Reference Centre for Complex Glomerular Diseases (CSUR), IdISSC, Madrid, Spain; Department of Medicine, Complutense University, Madrid, Spain; Nephrology Department, San Carlos Clinical University Hospital, National Reference Centre for Complex Glomerular Diseases (CSUR), IdISSC, Madrid, Spain; Department of Medicine, Complutense University, Madrid, Spain; Nephrology Department, Fundación Jiménez Díaz, Madrid, Spain; Nephrology Department, Fundación Jiménez Díaz, Madrid, Spain; Nephrology Department, Fundació Puigvert, Barcelona, Spain; Nephrology Department, Marqués de Valdecilla Hospital, Madrid, Spain; Nephrology Department, San Carlos Clinical University Hospital, National Reference Centre for Complex Glomerular Diseases (CSUR), IdISSC, Madrid, Spain; Nephrology Department, Villalba General University Hospital, Villalba, Spain; Nephrology Department, Gregorio Marañón General University Hospital, Madrid, Spain; Nephrology Department, Badajoz University Hospital, Badajoz, Spain; Nephrology and Kidney Transplantation Department, National Reference Centre for Complex Glomerular Diseases (CSUR), Hospital Clínic Barcelona–University of Barcelona, Barcelona, Spain; Nephrology Department, Germans Trias i Pujol Hospital, Badalona, Spain; Nephrology Department, Don Benito Hospital, Villanueva, Spain; Nephrology Department, Bellvitge University Hospital, Hospitalet de Llobregat, Spain; Nephrology Department, Consorci Sanitari de l’Anoia, Igualada, Spain; Nephrology Department, Miguel Servet University Hospital, Zaragoza, Spain; Nephrology Department, Canarias University Hospital, Tenerife, Spain; Nephrology Department, Vall d’Hebron University Hospital, National Reference Centre for Complex Glomerular Diseases (CSUR), RICORS 2040, Barcelona, Spain; Nephrology Department, San Carlos Clinical University Hospital, National Reference Centre for Complex Glomerular Diseases (CSUR), IdISSC, Madrid, Spain; Department of Medicine, Complutense University, Madrid, Spain

**Keywords:** CKD, IgA nephropathy, kidney biopsy, prognosis, proteinuria

## Abstract

**Background:**

Sparsentan has demonstrated significant antiproteinuric effects in randomized trials of IgA nephropathy (IgAN). However, real-world evidence remains scarce and is largely limited to short-term follow-up. Data on the durability of the antiproteinuric response in routine clinical practice are still lacking.

**Methods:**

This multicenter retrospective study included patients with biopsy-proven IgAN treated with sparsentan in routine clinical practice across Spanish centers. Patients were eligible if they initiated sparsentan outside a clinical trial and had at least 3 months of follow-up. The primary outcome was the proportion of patients achieving a >50% reduction in proteinuria at 3 months (responders). Secondary outcomes included changes in proteinuria and kidney function at 6 and 12 months, as well as safety.

**Results:**

A total of 41 patients were included. The median age was 42 years, with a median baseline estimated glomerular filtration rate (eGFR) of 44 ml/min/1.73 m^2^ and 95% receiving concomitant sodium–glucose cotransporter-2 (SGLT2) inhibitors. Median baseline proteinuria was 1163 mg/24 h. At 3 months, proteinuria decreased to 772 mg/24 h (median reduction 44.1%), with 51% of patients achieving a >50% reduction. Among patients with extended follow-up, the antiproteinuric effect was sustained at 6 months (median reduction 46.9%) and persisted at 12 months (median reduction 38.9%), despite increased variability. Notably, kidney function remained stable throughout follow-up, despite a moderately reduced baseline eGFR. No clinically relevant hyperkalemia or severe adverse events were observed.

**Conclusions:**

In this multicenter real-world Spanish cohort, sparsentan was associated with a sustained reduction in proteinuria in patients with biopsy-proven IgAN, maintained up to 12 months, along with preserved kidney function even in those with moderately reduced baseline eGFR, and a favorable safety profile. These findings support the effectiveness of sparsentan on top of contemporary standard of care and provide early evidence of sustained benefit in routine clinical practice.

KEY LEARNING POINTS **What was known:**

- Sparsentan reduces proteinuria in IgAN in randomized trials. However, real-world evidence is scarce and largely limited to short-term follow-up, with little information on durability of response under routine clinical conditions and contemporary background therapy.

**This study adds:**

- This multicenter real-world Spanish cohort provides the first long-term data up to 12 months, demonstrating a rapid antiproteinuric effect of sparsentan on top of optimized standard of care including SGLT2 inhibitors, with consistent response across sexes and stable kidney function over follow-up.

**Potential impact:**

- These data strengthen the role of sparsentan as an effective add-on therapy in IgAN, supporting its use in routine practice beyond trial populations and providing clinically relevant evidence on proteinuria reduction under contemporary treatment strategies.

## INTRODUCTION

Immunoglobulin A nephropathy (IgAN) is the most common primary glomerular disease worldwide and remains a leading cause of chronic kidney disease progression and kidney failure [1–3]. Despite optimized supportive care, including renin–angiotensin system (RAS) blockade and, more recently, sodium–glucose cotransporter-2 inhibitors (SGLT2i), a substantial proportion of patients continue to exhibit persistent proteinuria and progressive renal function decline [2, 3].

Proteinuria is a key modifiable risk factor and a validated surrogate endpoint in IgAN. Multiple observational studies and meta-analyses have shown that reductions in proteinuria are strongly associated with improved renal outcomes. Current therapeutic targets therefore aim for proteinuria levels below 0.5 g/day and ideally <0.3 g/day [4–6]. These findings have positioned proteinuria reduction as a central treatment goal in both clinical trials and routine practice [4–6].

In recent years, the therapeutic landscape of IgAN has evolved substantially [3, 7–9]. SGLT2i have demonstrated a reduction in the risk of kidney disease progression in patients with IgAN in subgroup analyses of large randomized trials [8, 9]. In parallel, targeted therapies such as budesonide have shown efficacy in reducing proteinuria and preserving kidney function [10, 11], although their use remains limited to selected patients. Other approaches, including systemic corticosteroids, have also shown efficacy in high-risk patients, albeit at the cost of increased toxicity [12].

Sparsentan, a dual endothelin type A and angiotensin II receptor antagonist, represents a novel nonimmunosuppressive approach targeting both hemodynamic and inflammatory pathways [7, 13, 14]. In the phase 3 PROTECT trial, sparsentan achieved a significantly greater reduction in proteinuria compared with irbesartan at 36 weeks, with sustained effects over 2 years and a slower rate of estimated glomerular filtration rate (eGFR) decline [15]. Subsequent analyses have further highlighted the clinical relevance of achieving greater reductions in proteinuria, linking this response to a lower risk of kidney disease progression and improved long-term renal outcomes. [6, 16]. However, real-world evidence on sparsentan remains limited. Available data are restricted to small observational cohorts and case series, generally focused on early proteinuria changes and with short follow-up periods. The series reported by Schanz *et al*. showed a marked reduction in proteinuria at early time points, while small case series have reported similar findings in heterogeneous populations [17, 18]. Data on medium-term outcomes, predictors of response, and safety in patients receiving contemporary background therapy remain scarce.

In this context, we aimed to evaluate the early antiproteinuric response to sparsentan, and assess its medium-term efficacy and safety in a multicenter real-world cohort of patients with IgAN treated under current standards of care.

## MATERIALS AND METHODS

### Study design and population

This was a retrospective, multicenter observational study including patients with biopsy-proven IgAN treated with sparsentan in routine clinical practice across 14 Spanish centers.

**Figure 1:**
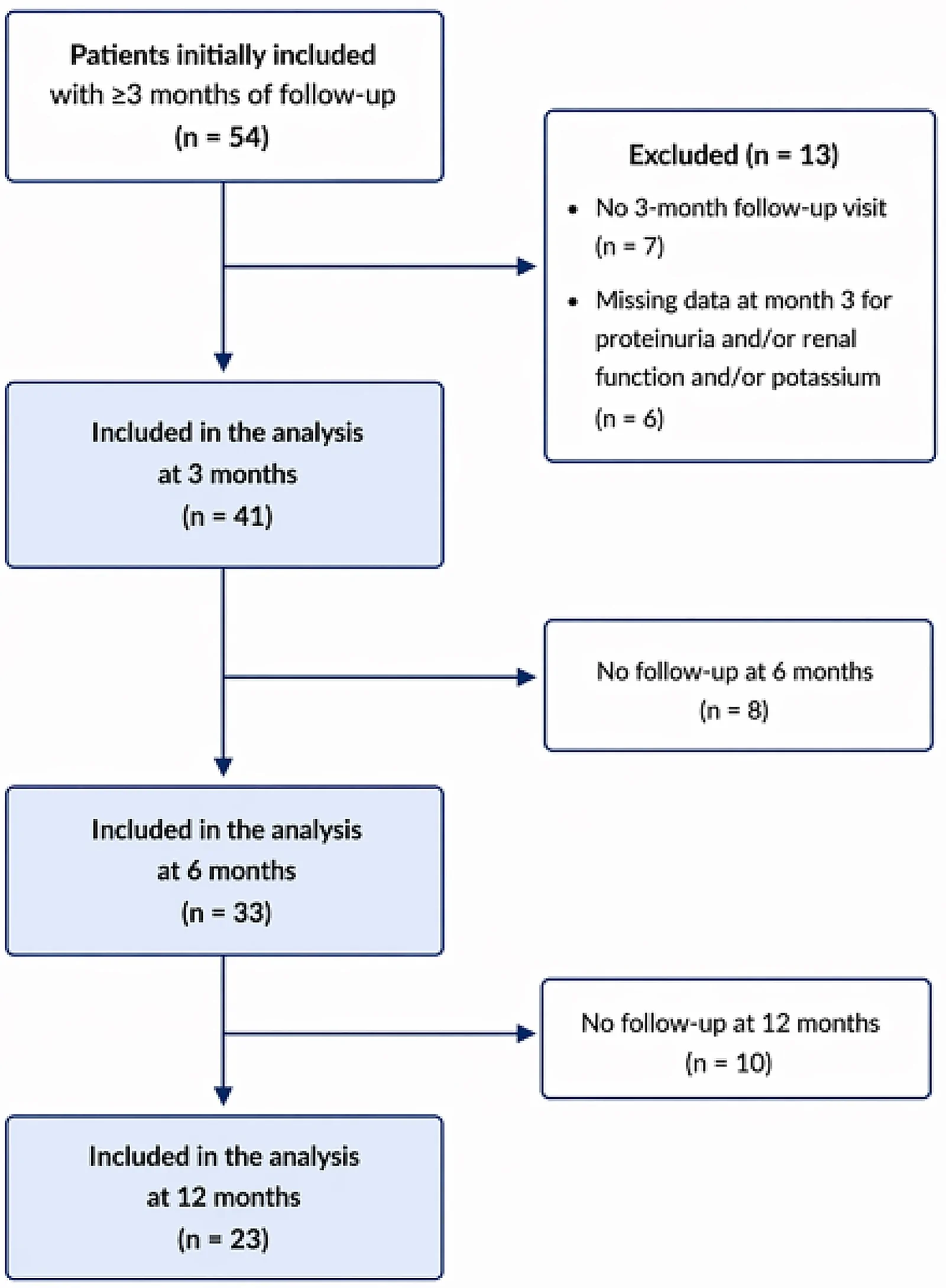
Patient flow chart diagram. Flow diagram of patient selection and follow-up. A total of 54 patients with at least 3 months of follow-up were initially considered. Thirteen patients were excluded due to absence of a 3-month follow-up visit (*n* = 7) or missing data on proteinuria, renal function, and/or potassium at 3 months (*n* = 6). A total of 41 patients were included in the 3-month analysis. Of these, 33 had available follow-up at 6 months (*n* = 8 without 6-month follow-up), and 23 had available follow-up at 12 months (*n* = 10 without 12-month follow-up).

Patients were eligible if they initiated treatment with sparsentan outside the context of a clinical trial and had at least 3 months of available follow-up data. A longitudinal subgroup analysis was performed in patients with available follow-up at 6 and 12 months (see Fig. 1). The study was conducted in accordance with the principles of the Declaration of Helsinki and was approved by the Ethics Committee of Hospital Clínico San Carlos.

### Data collection

Baseline data included demographic characteristics, comorbidities, laboratory parameters, and prior treatments. All patients had received optimized RAS blockade at the maximum tolerated dose before initiation of sparsentan. Concomitant treatment with SGLT2i at baseline was recorded. Clinical and laboratory data were collected from routine follow-up visits. Proteinuria, serum creatinine, and eGFR were recorded at baseline and at 1, 3, 6, and 12 months when available. Due to the observational design, follow-up time points were not uniform across all patients. Hematuria was recorded both as a categorical variable (presence/absence) and as a continuous variable when quantitative data were available. In centers reporting hematuria as a range of red blood cells per high-power field, the midpoint of the reported range was used for analysis. The predefined visit windows were ±21 days for the 3-month visit and ±30 days for the 6- and 12-month visits.

### Outcomes

The primary outcome was the proportion of patients achieving a reduction in proteinuria >50% at 3 months. Patients meeting this criterion were classified as responders. The >50% reduction in proteinuria used to define response was selected because it corresponded to the median proteinuria reduction observed in our cohort at 3 months. In addition, this threshold lies between the reduction reported in the PROTECT trial (∼50%) and the ∼60% reduction described at 14 weeks in the only available real-world sparsentan cohort, providing a clinically meaningful and pragmatic definition of response.

Secondary outcomes included changes in proteinuria at 6 and 12 months, evolution of kidney function over time, changes in blood pressure during follow-up, and safety outcomes, including hyperkalemia, acute kidney injury (AKI), and liver enzyme abnormalities.

### Statistical analysis

Continuous variables are presented as median and interquartile range (IQR), and categorical variables as counts and percentages. Paired comparisons between baseline and follow-up values were performed using the Wilcoxon signed-rank test. Comparisons between responders and nonresponders were performed using the Mann–Whitney U test for continuous variables and the χ^2^ test for categorical variables. To identify donor factors possibly associated with a decrease of proteinuria >50% at 3 months, we conducted a univariate analysis including the main clinical variables. A logistic regression model was constructed adjusted by backward stepwise regression based on maximum likelihood estimators including variables showing a *P* < .15 in the univariate analysis. Odds ratios and their significance were calculated for each variable according to criteria for entry (*P* < .05) and removal (*P* < .10). Possible interactions were assessed by introducing multiplicative terms. A *P*-value <.05 was considered statistically significant. All statistical analyses were performed using JASP statistical software (version 0.95.4; JASP Team).

## RESULTS

### Baseline characteristics

A total of 54 patients were initially considered for inclusion in the study. Thirteen were excluded due to incomplete or inconsistent follow-up data. A total of 41 patients were included in the analysis at 3 months. Only four patients discontinued sparsentan during the first year of follow-up. Therefore, the progressive reduction in the number of patients available for analysis between months 3 and 12 was mainly related to limited follow-up duration rather than treatment discontinuation (Fig. 1). Baseline characteristics are summarized in Tables 1 and 2.

**Table 1:** Baseline characteristics.

	*n* = 41, month 3
Age at sparsentan initiation, years (median, IQR)	42 (36–54)
Women (*n*, %)	16 (39%)
Ethnicity: - Caucasian - Black - Latin - Asian	28 (68.3%) 0 10 (24.4%) 3 (7.3%)
Hypertension yes (*n*, %)	23 (56.1%)
DM yes (*n*, %)	2 (4.9%)
Time biopsy to sparsentan initiation, months (median, IQR)	16 (9–49)
Kidney biopsy Oxford score M^a^: 0 (*n*, %) 1 (*n*, %)	23 (56.1%) 14 (34.1%)
Kidney biopsy Oxford score E^a^: 0 (*n*, %) 1 (*n*, %)	21 (51.2%) 16 (39%)
Kidney biopsy Oxford score S^a^: 0 (*n*, %) 1 (*n*, %)	12 (29.3%) 25 (61%)
Kidney biopsy Oxford score T^a^: 0 (*n*, %) 1 (*n*, %) 2 (*n*, %)	20 (48.8%) 16 (39%) 1 (2.4%)
Kidney biopsy Oxford score C^a^: 0 (*n*, %) 1 (*n*, %) 2 (*n*, %)	23 (56.1%) 13 (31.7%) 1 (2.4%)
Previous maximum tolerated ACEi/ARA II (*n*, %)	41 (100%)
SGLT2i at the time of sparsentan initiation (*n*, %)	39 (95.1%)

a In four patients, Oxford score was not performed.

ACEi, angiotensin-converting enzyme inhibitors; ARA II, angiotensin II receptor antagonist; C, cellular/fibrocellular crescents; DM, diabetes mellitus; E, endocapillary hypercellularity; M, mesangial hypercellularity; S, segmental sclerosis; T, tubular atrophy/interstitial fibrosis.

**Table 2:** Univariate analysis, differences in clinical parameters at baseline, 1 month, 6 months, and 12 months after sparsentan initiation.

Variable	3-month follow-up			6-month follow-up			12-month follow-up		
	Baseline (*n* = 41)	Month 3	*P*	Baseline (*n* = 33)	Month 6	*P*	Baseline (*n* = 23)	Month 12	*P*
Patients receiving 400 mg dose, *n* (%)	–	37 (90.2%)	–	–	29 (87.9%)	–	–	19 (82.6%)	–
Creatinine (mg/dl), median (IQR)	1.65 (1.30–2.10)	1.72 (1.35–2.08)	.334	1.65 (1.28–2.10)	1.77 (1.30–2.10)	.201	1.65 (1.45–2.05)	1.83 (1.43–2.07)	.284
eGFR (ml/min/1.73 m^2^), median (IQR)	44 (33–61)	41 (33–64)	.940	44 (33–65.25)	43 (33–69)	.540	40 (33.7–52.5)	40 (34–59.5)	.433
Potassium (mEq/l), median (IQR)	4.7 (4.3–4.9)	4.5 (4.4–4.8)	.754	4.7 (4.3–4.8)	4.65 (4.2–4.9)	.724	4.5 (4.3–4.8)	4.5 (4.4–4.7)	.643
ALT (U/l), median (IQR)	20 (14–28.5)	20 (14–28.5)	.196	20 (14.75–30)	15.5 (13.25–21)	.079	17 (14–26.5)	17.5 (15–22.7)	.230
AST (U/l), median (IQR)	20 (17–27.5)	20 (17–27.5)	.626	20 (17.25–26.75)	19 (15.5–23)	.220	20 (17.5–31)	18 (15–28.2)	.065
Hemoglobin (g/dl), median (IQR)	14.7 (13–16)	14.3 (12.2–15.7)	.003	14.7 (12.8–16)	13.6 (12–15.5)	.198	14.7 (13–16.1)	14.4 (12.9–16.7)	.833
Presence of hematuria, *n* (%)	20 (48.8%)	2 (4.8%)	.025	17 (51.5%)	8 (24.2%)	.124	17 (73.9%)	11 (47.8%)	.127
Hematuria (RBCs/HPF), median (IQR)	5 (5–17.5)	6.5 (0–13)	.043	5 (4–17.5)	5 (0–20)	.003	5 (5–9)	5 (0–7.5)	.106
UACR (mg/g), median (IQR)	884 (686–1204)	401 (176.5–785)	<.001	917 (754–1246)	354 (182–637)	<.001	950 (740–1274)	561.5 (182.2–862.8)	.002
UACR reduction (%), median (IQR)	–	50.4 (31.3–75.7)	–	–	51.1 (30.9–75.5)	–	–	45.6 (−6.7–83.4)	–
Proteinuria (mg/24 h), median (IQR)	1163 (1068–1743)	772 (557–1140)	<.001	1197 (1082–1603)	760 (478–1139)	<.001	1310 (1080–1640)^a^	800 (385–1479)	.073
Proteinuria reduction (%), median (IQR)	–	44.1 (21.9–66.9)	–	–	46.9 (24.9–67.1)	–	–	38.9 (−25.3–73.7)	–
SBP (mmHg), median (IQR)	120 (115.5–129.5)	120 (110–121)	.035	120 (115.5–125)	125 (110–130)	.624	120 (115–125)	120 (110–124.3)	.754
DBP (mmHg), median (IQR)	71 (70–80.5)	70 (63–75)	.083	70 (69–78)	71 (66.5–79.8)	.072	70 (70–77)	70 (65–77.2)	.553

B, baseline; DBP, diastolic blood pressure; Hb, hemoglobin; HPF, high-power field; K, potassium; RBCs, red blood cells; SBP, systolic blood pressure; UACR, urinary albumin-to-creatinine ratio.

At the time of sparsentan initiation, the median age was 42 years (IQR 36–54), and 39% were women. Hypertension was present in a substantial proportion of patients, while diabetes was uncommon. Median baseline proteinuria was 1163 mg/24 h (IQR 1068–1743), and median eGFR was 44 ml/min/1.73 m^2^ (IQR 33–61). Baseline hematuria was highly prevalent and present in the 48.8% of patients consistent with active urinary sediment. Baseline clinical data are summarized in Tables 1 and 2. When stratified by sex, no significant differences were observed in baseline proteinuria, eGFR, or prevalence of hematuria at baseline.

Kidney biopsy diagnoses were available in all patients but the Oxford classification score was unavailable for four patients. The median time from kidney biopsy to initiation of sparsentan was 16 months (IQR 8–49), reflecting a cohort with variable disease duration prior sparsentan initiation and the heterogeneity inherent to routine clinical practice. According to the Oxford classification, segmental sclerosis (S1) was frequently observed (61%), while endocapillary hypercellularity (E1, 39%), mesangial hypercellularity (M1, 34.1%), and tubulointerstitial lesions (T score) were less consistently present, reflecting a heterogeneous histological profile (Table 1).

**Figure 2:**
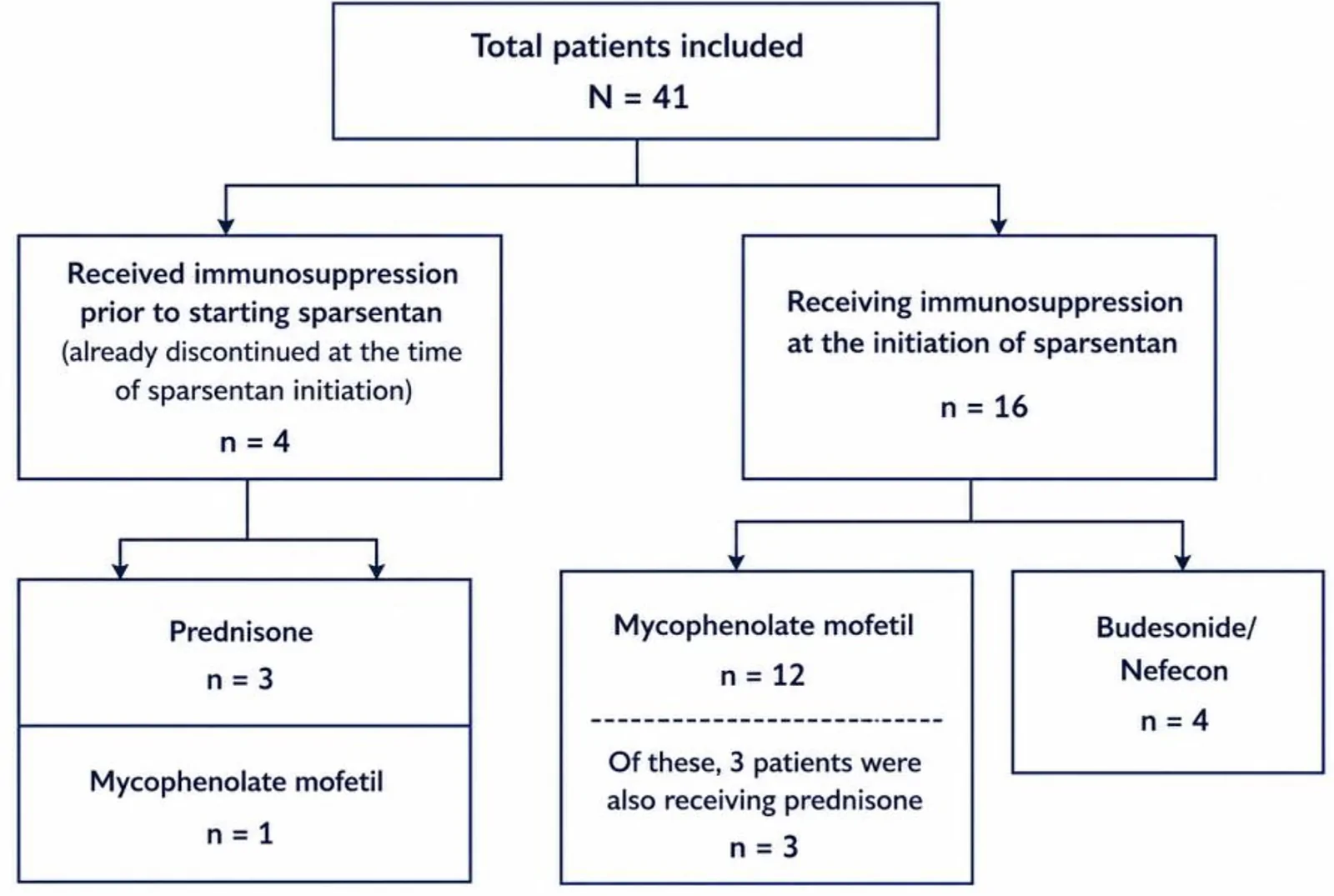
Immunosuppressive therapy before and at the time of sparsentan initiation.

All patients had previously received optimized RAS blockade at the maximum tolerated dose before initiation of sparsentan. At baseline, 95% of patients were receiving SGLT2i, reflecting contemporary standard of care. At the time of sparsentan initiation, patients had been receiving SGLT2 inhibitors for a median of 16 (IQR 9–25) months and RAS inhibitors for a median of 43 (IQR 21–85) months. Four patients had received previous immunosuppressive therapy before sparsentan initiation, and 16 were receiving concomitant immunosuppressive treatment at baseline. Among the latter, the median time from immunosuppressive therapy initiation to sparsentan initiation was 7.8 months (IQR 5.3–19.0). Details of immunosuppressive therapy are summarized in Fig. 2. All 16 patients receiving immunosuppressive therapy at baseline were included in the 3-month analysis; 15 and 11 of these patients were included in the 6- and 12-month analyses, respectively, according to the available duration of follow-up. Baseline characteristics were also compared between patients receiving concomitant immunosuppressive therapy at the time of sparsentan initiation and those who were not. No significant differences were observed between the two groups (Table 3).

**Table 3:** Baseline characteristics and 3-month outcomes according to concomitant immunosuppressive therapy at sparsentan initiation.

	Patients on immunosuppression at sparsentan initiation (*n* = 16)	Patients not on immunosuppression at sparsentan initiation (*n* = 25)	*P*
Age at sparsentan initiation, years (median, IQR)	40 (36.2–51.2)	42 (36–59)	.521
Women (*n*, %)	7 (43.7%)	9 (36%)	.614
Time biopsy to sparsentan initiation, months (median, IQR)	25 (13–54.7)	12 (6–28)	.239
Baseline creatinine (mg/dl) (median, IQR)	1.75 (1.45–2.2)	1.57 (1.3–1.98)	.454
Baseline eGFR (ml/min/1.73 m^2^)	37.5 (35.2–55.5)	47.5 (33–69)	.580
Presence of hematuria, yes (*n*, %)	13 (81.2%)	19 (76%)	.692
Baseline hematuria RBCs per high-power field (median, IQR)	5 (3–25)	8.5 (5–15)	.872
Baseline proteinuria, mg/24 h (median, IQR)	1352 (1097–2508)	1101 (966–1551)	.191
SBP, mmHg (median, IQR)	122 (110–130)	120 (117–129)	.931
DBP, mmHg (median, IQR)	74 (67–87)	70.5 (70–77.5)	.965
Month 3			
Creatinine (mg/dl), median (IQR)	1.90 (1.31–2.19)	1.62 (1.35–2.00)	.316
eGFR (ml/min/1.73 m^2^), median (IQR)	37.5 (32.3–60.5)	48.4 (33–64)	.329
Proteinuria (g/24 h), median (IQR)	812 (664–1070)	700 (558–1170)	.533
% Decrease proteinuria (baseline-month 3), median (IQR)	44.7 (26.7–65.4)	44.1 (21.9–73.2)	.924
Proteinuria decrease >50%, yes (*n*, %)	7 (43.7%)	14 (56%)	.567
Presence of hematuria, yes (*n*, %)	9 (56.2%)	13 (52%)	.622
Hematuria, RBCs per high-power field (median, IQR)	10 (3–17)	5 (0–10)	.417
SBP, mmHg (median, IQR)	120 (110–127)	118.5 (110–121)	.886
DBP, mmHg (median, IQR)	76 (73–81)	70 (63–75)	.033

RBCs, red blood cells; SBP, systolic blood pressure; DBP, diastolic blood pressure.

To assess the potential impact of incomplete follow-up on the longitudinal analyses, baseline characteristics were compared between patients with and without available 12-month follow-up (Table 4). No clinically relevant differences were observed between groups.

**Table 4:** Baseline characteristics of patients with and without 12-month follow-up.

	Patients with 12-months follow-up (*n* = 23)	Patients without 12-months follow-up (*n* = 18)	*P*
Age at sparsentan initiation, years (median, IQR)	45 (38.5–52.5)	38 (34.5–56)	.520
Women (*n*, %)	10 (43.5%)	6 (33.3%)	.509
Time biopsy to sparsentan initiation, months (median, IQR)	16 (9.5–41)	24 (9–65.7)	.478
Creatinine (mg/dl) (median, IQR)	1.65 (1.45–2.05)	1.63 (1.28–2.1)	.958
eGFR (ml/min/1.73 m^2^)	40 (33.7–52.5)	49.5 (34.2–69)	.341
Presence of hematuria, yes (*n*, %)	18 (78.3%)	14 (77.8%)	.970
Hematuria RBCs per high-power field (median, IQR)	5 (5–9)	10 (5.7–20)	.166
UACR, mg/g creatinine (median, IQR)	950 (740–1274)	862 (670–982)	.490
Proteinuria, mg/24 h (median, IQR)	1310 (1080–1614)	1101 (1067–1900)	.908
SBP, mmHg (median, IQR)	120 (115–125)	127 (117–130)	.288
DBP, mmHg (median, IQR)	70 (70–77)	74.5 (70–85)	.208
Immunosuppression at the time of sparsentan initiation (*n*, %)	11 (47.8%)	5 (27.8%)	.377

DBP, diastolic blood pressure; RBCs, red blood cells; SBP, systolic blood pressure; UACR, urinary albumin-to-creatinine ratio.

### Early response at 3 months

**Figure 3:**
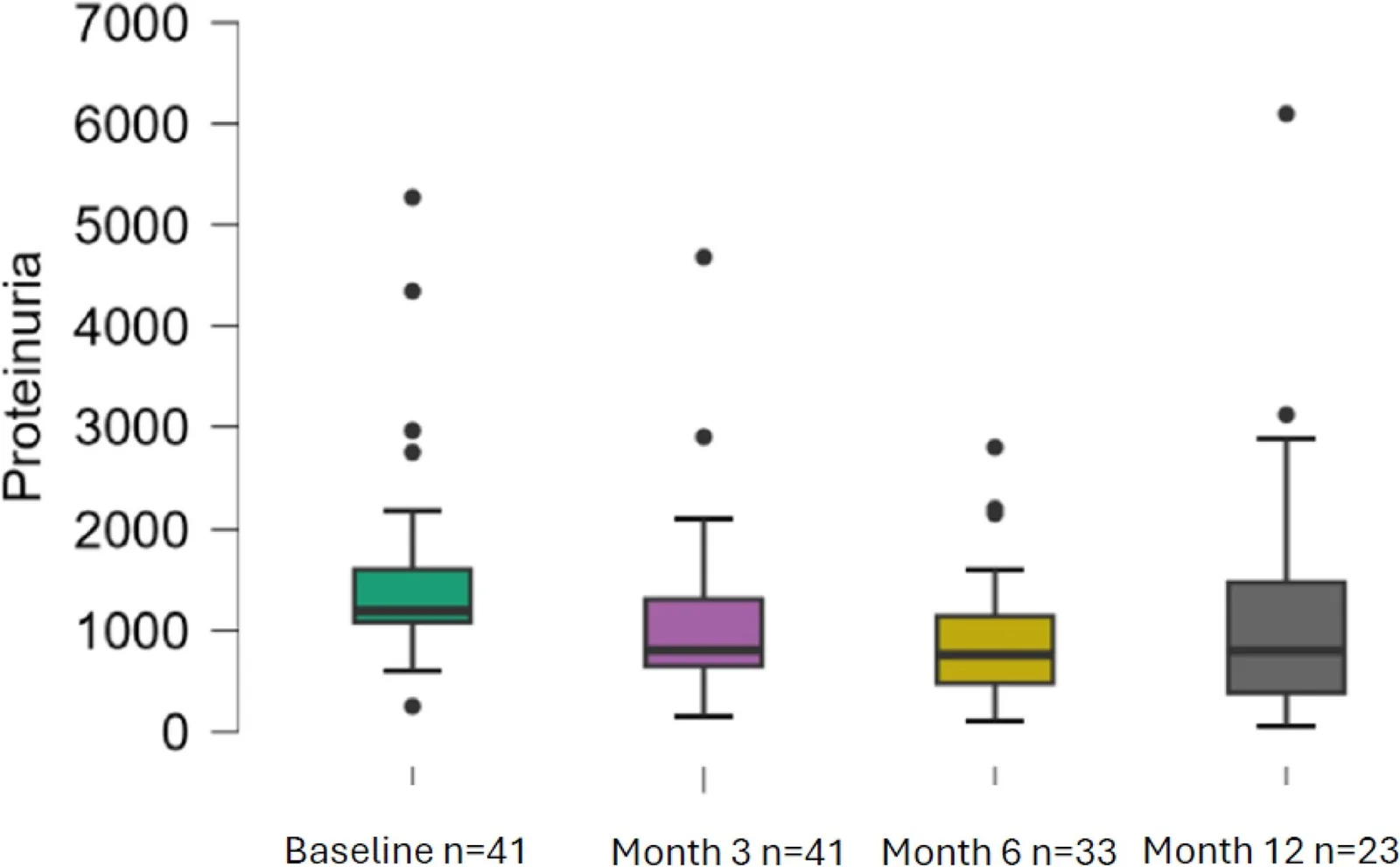
Evolution of proteinuria following sparsentan initiation. Boxplots represent median values and IQRs of proteinuria (g/24 h) at baseline, and at 3, 6, and 12 months of follow-up. Whiskers indicate the range within 1.5 times the IQR, and dots represent outliers. A progressive reduction in proteinuria is observed over time, with an early decrease at 3 months that is sustained at 6 and 12 months.

At 3 months, a significant and clinically meaningful reduction in proteinuria was observed. Median proteinuria decreased from 1163 mg/24 h at baseline to 772 mg/24 h (IQR 557–1140; *P* < .01), corresponding to a median relative reduction of 44.1% (IQR 21.9–66.99) (Table 2 and Fig. 3). Overall, 51% of patients achieved a reduction in proteinuria >50% (Table 5). At month 3, all but two patients showed a reduction in proteinuria, with the smallest reduction being 11%. Kidney function remained stable during this early phase, with no significant differences in eGFR compared with baseline values. A small although statistically significant reduction in systolic blood pressure was observed (120 [IQR 115.5–129.5] vs 120 [IQR 110–121] mmHg, *P* = .035), which was not considered clinically meaningful. No significant change was observed in diastolic blood pressure (71 [IQR 70–80.5] vs 70 [IQR 63–75] mmHg, *P* = .083) (Table 2).

**Table 5:** Clinical variables related with proteinuria decrease >50% at month 3. Univariate analysis.

	Nonresponders: decrease ≤50% *n* = 19	Responders: decrease >50% *n* = 21	*P*
Sex (female), *n* (%)	8	7	.567
Age (years), median (IQR)	42 (37–50)	48 (33.5–55.5)	.903
Hypertension (yes), *n* (%)	10 (52.6%)	12 (57.1%)	.775
Kidney biopsy Oxford score M^a^ 0 (yes), *n* (%)	14 (73.7%)	9 (42.8%)	.083
Kidney biopsy Oxford score E^a^ 0 (yes), *n* (%)	10 (52.6%)	10 (47.6%)	.999
Kidney biopsy Oxford score S^a^ 0 (yes), *n* (%)	7 (36.8%)	5 (23.8%)	.480
Kidney biopsy Oxford score T^a^ 0 (yes), *n* (%)	12 (63.1%)	7 (33.3%)	.102
Kidney biopsy Oxford score C^a^ 0 (yes), *n* (%)	11 (57.9%)	11 (52.4)	.584
Baseline eGFR (ml/min/1.73 m^2^), median (IQR)	43 (34.5–66.5)	45 (35.2–53.7)	.910
Baseline eGFR > 45 ml/min/1.73 m^2^ yes, (*n*, %)	9 (47.4%)	10 (47.6%)	.987
Presence of hematuria at sparsentan initiation (yes), *n* (%)	12 (63.1%)	20 (95.2%)	.011
RBCs per high-power field at sparsentan initiation (median, IQR)	5 (3–10)	10 (5–30)	.271
Baseline proteinuria (g/24 h), median (IQR)	1191 (1081–1614)	1223 (1045–2321)	.641
Baseline UACR (mg/g), median (IQR)	950 (796–1052)	865 (706.8–1379)	.951
Maximum tolerated ACE inhibitor/ARB dose prior to sparsentan initiation, yes (*n*, %)	16 (84.2%)	16 (76.2%)	.527
eGFR month 3 (ml/min/1.73 m^2^), median (IQR)	41 (32–67.5)	42 (35–59)	.892
SBP month 3 (mmHg), median (IQR)	120 (110–127.3)	114.5 (100–120)	.065
DBP month 3 (mmHg), median (IQR)	74 (70–80.25)	70 (63–75.2)	.113
% decrease SBP month 3	3.8 (−9.0–6.5)	6–02 (1.46–14.8)	.069
% decrease DBP month 3	0 (−7.0–6.2)	9.8 (1.8–16.6)	.017
% proteinuria reduction (median, IQR)	68 (58.5–78.1)	21.7 (15.4–37.1)	<.001

ARB, angiotensin receptor blockers; ACE, angiotensin-converting enzyme; C, cellular/fibrocellular crescents; DBP, diastolic blood pressure; E, endocapillary hypercellularity; M, mesangial hypercellularity; RBCs, red blood cells; S, segmental sclerosis; SBP, systolic blood pressure; T, tubular atrophy/interstitial fibrosis; UACR, urinary albumin-to-creatinine ratio.

When stratified according to response, defined by proteinuria reduction >50%, baseline hematuria was more frequently observed among responders than nonresponders (95.2% vs 63.1%; *P* = .011). Compared with nonresponders, patients achieving a >50% reduction in proteinuria showed a greater relative reduction in diastolic blood pressure (9.8% [IQR 1.8–16.6] vs 0% [IQR −7.0 to 6.2], *P* = .017) and a trend toward a greater reduction in systolic blood pressure (6.02% [IQR 1.46–14.8] vs 3.8% [IQR −9.0 to 6.5], *P* = .069) (Table 5). No differences were observed between groups in baseline proteinuria, eGFR, or other clinical characteristics (Table 5). In multivariate analysis, no variables reached statistical significance, although the absence of hematuria showed a trend toward a lower likelihood of response (Table 6).

**Table 6:** Factors associated with >50% proteinuria reduction at 3 months: exploratory multivariable logistic regression analysis.

	OR	95% CI	*P*
Baseline eGFR (ml/min/1.73 m^2^),	1.032	0.979–1.008	.240
Baseline proteinuria (g/24 h)	1.000	0.999–1.001	.644
Hematuria at sparsentan initiation	8.471	0.416–172.608	.165
% decrease SBP month 3	0.891	0.766–1.035	.131
% decrease DBP month 3	0.851	0.724–1.001	.052

CI, confidence interval; DBP, diastolic blood pressure; OR, odds ratio; SBP, systolic blood pressure.

Using proteinuria <0.5 g/24 h as an alternative definition of treatment response yielded results consistent with those obtained using a >50% reduction in proteinuria. A threshold of <1 g/24 h was not evaluated because the median baseline proteinuria was already ∼1 g/24 h, limiting its ability to discriminate treatment response.

When analyzed according to sex, women presented a higher prevalence of hematuria at baseline and lower systolic blood pressure compared with men. These differences persisted 3 months after initiation of sparsentan. Despite lower baseline values, the relative reduction in systolic blood pressure was similar between both groups. In contrast, no differences were observed between men and women in baseline proteinuria or in the magnitude of proteinuria reduction at 3 months. Kidney function was also comparable between both groups, with no differences in eGFR at baseline or during follow-up (Table 7).

**Table 7:** Sex-related differences at baseline and at month 3 after sparsentan initiation (*n* = 41).

	Women (*n* = 16)	Men (*n* = 25)	*P*
Baseline			
Age at sparsentan initiation, years (median, IQR)	39 (34.5–48.2)	48 (37–65)	.109
Hypertension yes (*n*, %)	8 (50%)	15 (60%)	.523
DM yes (*n*, %)	0	2	.492
Time biopsy to sparsentan initiation, months (median, IQR)	13 (9.5–22.7)	25 (9–62)	.303
Baseline creatinine (mg/dl), median (IQR)	1.38 (1.04–1.79)	1.71 (1.53–2.12)	.053
Baseline eGFR (ml/min/1.73 m^2^), median (IQR)	52 (36–74)	42 (33–53)	.378
Baseline proteinuria (g/24 h), median (IQR)	1219 (1081–1698)	1118 (966–1757)	.999
Baseline albuminuria (UACR), median (IQR)	900 (737.5–1136)	868 (46–1251)	.964
RBCs per high-power field at sparsentan initiation (median, IQR)	20 (5–47.5)	5 (4.5–10)	.024
Presence of hematuria at sparsentan initiation (yes), *n* (%)	12	20	.706
SBP (mmHg), median (IQR)	117.5 (110–123.5)	125 (120–130)	.050
DBP (mmHg), median (IQR)	70 (67–76.25)	75 (70–85)	.083
Kidney biopsy Oxford score M^a^: 0 (*n*, %) 1 (*n*, %)	12 3	11 11	.065
Kidney biopsy Oxford score E^a^: 0 (*n*, %) 1 (*n*, %)	7 8	14 8	.306
Kidney biopsy Oxford score S^a^: 0 (*n*, %) 1 (*n*, %)	5 10	7 15	
Kidney biopsy Oxford score T^a^: 0 (*n*, %) 1 (*n*, %) 2 (*n*, %)	10 5 0	10 11 1	.923
Kidney biopsy Oxford score C^a^: 0 (*n*, %) 1 (*n*, %) 2 (*n*, %)	7 7 1	16 6 0	.183
Previous maximum tolerated ACEi/ARB (*n*, %)	16	25	
SGLT2i at the time of sparsentan initiation (*n*, %)	15	24	.744
Month 3			
Patients on 400 mg dose	14	23	.636
Creatinine (mg/dl), median (IQR)	1.48 (1.01–1.74)	1.93 (1.49–2.13)	.018
eGFR (ml/min/1.73 m^2^), median (IQR)	55 (33.7–71.4)	39 (31–59)	.131
Proteinuria (g/24 h), median (IQR)	782 (619–1013)	772 (540–1258)	.778
% Decrease proteinuria, median (IQR)	47.7 (19.6–71)	41.4 (27.2–65.4)	.924
Proteinuria decrease >50%	7	14	.567
Albuminuria (UACR, mg/g creatinine), median (IQR)	427 (222–603)	347 (141–918)	.810
% Decrease albuminuria, median (IQR)	52.6 (20.4–75.8)	48.2 (34.8–68.9)	.885
RBCs per high-power field (median, IQR)	12 (5.2–31)	5 (0–10)	.036
SBP month 3 (mmHg), median (IQR)	110 (100–120)	120 (111.5–127)	.034
DBP month 3 (mmHg), median (IQR)	70 (64–75)	71.5 (70–80.2)	.231
% decrease SBP month 3	7.4 (0–13.8)	3.8 (0.4–6.5)	.398
% decrease DBP month 3	5.2 (−3.3–12.3)	2.4 (−1.1–12.7)	.953

DM, diabetes mellitus; RBCs, red blood cells; UACR, urinary albumin-to-creatinine ratio; SBP, systolic blood pressure; DBP, diastolic blood pressure; ARB, angiotensin receptor blockers; ACEi, angiotensin-converting enzyme inhibitors; C, cellular/fibrocellular crescents; E, endocapillary hypercellularity; M, mesangial hypercellularity; S, segmental sclerosis; T, tubular atrophy/interstitial fibrosis.

Response was also evaluated according to concomitant immunosuppressive therapy at the time of sparsentan initiation. No significant differences were observed between patients receiving and not receiving immunosuppressive therapy in proteinuria reduction at 3 months, the proportion achieving a >50% reduction in proteinuria, kidney function, or persistence of hematuria (Table 3).

### Medium term outcomes (6 and 12 months)

Thirty-three patients had available follow-up at 6 months, of whom 23 also had follow-up at 12 months, representing a longitudinal subset of the initial cohort.

At 6 months, the reduction in proteinuria was sustained, with median values decreasing from 1197 mg/24 h (IQR 1082–1603) to 760 mg/24 h (IQR 478–1139; *P* < .001), corresponding to a median reduction of 46.9% (IQR 24.9–67.1). The magnitude of reduction at this time point was comparable to that observed at 3 months, suggesting an early stabilization of the antiproteinuric effect.

Among patients with longer follow-up, this effect persisted at 12 months. Median proteinuria was 800 mg/24 h (IQR 385–1479), corresponding to a median reduction of 38.9% (IQR −25.3 to 73.7). Although greater variability was observed at this stage, likely reflecting the smaller sample size and differences in follow-up duration, the overall antiproteinuric effect was maintained. These data are summarized in Fig. 2 and Table 2. At 12 months, five patients (21.7%) had proteinuria <0.5 g/24 h and three patients (13%) had proteinuria <0.3 g/24 h.

To further contextualize the evolution of kidney function, eGFR during the 12 months preceding sparsentan initiation was compared with the subsequent 12-month follow-up in the same cohort of patients who completed 12 months of follow-up and had available eGFR data at all three time points (3, 6, and 12 months). Median eGFR declined significantly from 47 (36–74) to 40 (33.7–52.5) ml/min/1.73 m^2^ during the year before sparsentan initiation (*P* = .003). In contrast, no significant change was observed between baseline and 12 months after sparsentan initiation (40 [33.7–52.5] vs 40 [34–59.5] ml/min/1.73 m^2^; *P* = .433), despite the marked reduction in proteinuria observed during follow-up.

Albuminuria followed a parallel trajectory, with sustained reductions over time. No relevant changes were observed in serum creatinine or eGFR at either 6 or 12 months. At 6 months, systolic and diastolic blood pressure remained unchanged compared with baseline (120 [IQR 115.5–125] vs 125 [IQR 110–130] mmHg, *P* = .624, and 70 [IQR 69–78] vs 71 [IQR 66.5–79.8] mmHg, *P* = .072, respectively). Similarly, no significant changes were observed at 12 months (120 [IQR 115–125] vs 120 [IQR 110–124.3] mmHg, *P* = .754, and 70 [IQR 70–77] vs 70 [IQR 65–77.2] mmHg, *P* = .553, respectively).

### Safety

Sparsentan was generally well tolerated during follow-up. Serum potassium levels remained stable overall. Mild hyperkalemia (potassium >5.5 mmol/l) was observed in four patients, but none developed potassium levels ≥6.0 mmol/l. No episodes required treatment discontinuation, and no clinically relevant complications related to hyperkalemia were recorded. Total five episodes of AKI were documented. According to Kidney Disease: Improving Global Outcomes criteria, four episodes were classified as stage 1 and one episode as stage 2, with no stage 3 AKI events observed. All episodes were transient, with recovery of baseline kidney function in all cases and no requirement for renal replacement therapy. In four patients, up-titration to the target dose of 400 mg was not feasible, and treatment was maintained at 200 mg once daily due to hypotension.

Four patients discontinued sparsentan during the first year of treatment. One patient discontinued sparsentan because of worsening heart failure. The patient had pre-existing heart failure and, following clinical decompensation with New York Heart Association class II–III symptoms, initiation of a mineralocorticoid receptor antagonist was considered clinically indicated by the treating cardiologist, leading to discontinuation of sparsentan. One patient discontinued sparsentan after developing stage 2 AKI. The treating nephrologist decided to discontinue sparsentan based on the overall clinical assessment. Two patients discontinued sparsentan because of persistent liver enzyme elevation. In the first patient, liver function tests showed aspartate aminotransferase (AST) 77 U/l and alanine aminotransferase (ALT) 123 U/l. The sparsentan dose was reduced to 200 mg/day, but treatment was discontinued because of persistent abnormalities. At the time of discontinuation, ALT had normalized (29 U/l), whereas AST remained mildly elevated (96 U/l) and gamma-glutamyl transferase (GGT) was 78 U/l. In the second patient, AST and ALT increased to 106 and 286 U/l, respectively, while alkaline phosphatase, GGT, bilirubin, coagulation parameters, and albumin remained within the normal range. Pitavastatin and allopurinol were discontinued, but repeat testing showed further elevation of AST (146 U/l) and ALT (356 U/l), with all other liver parameters remaining normal. An extensive hepatological work-up was negative, and liver biopsy findings were compatible with drug-induced liver injury.

No deaths or progression to end-stage kidney disease was observed during the study period.

## DISCUSSION

In this multicenter real-world cohort, treatment with sparsentan was associated with a rapid and clinically meaningful reduction in proteinuria that was already evident at 3 months, remained stable at 6 months, and persisted at 12 months in patients with longer follow-up. The overall pattern is not one of progressive decline but rather of an early response followed by stabilization, which likely reflects how this therapy behaves in routine clinical practice and may be more informative than isolated time-point comparisons.

The magnitude of the antiproteinuric response observed in our cohort is consistent with that reported in the PROTECT trial, where sparsentan achieved reductions in proteinuria in the range of 50% [15]. In our series, more than half of the patients achieved a reduction in proteinuria >50% at 3 months, despite a baseline proteinuria above 1 g/24 h. When considered alongside the PROTECT population, in which baseline proteinuria was higher, in the range of ∼1.8–2.0 g/24 h, these findings suggest that the degree of response is preserved across different baseline risk profiles. Importantly, this sustained reduction in proteinuria was accompanied by stable kidney function over time in a cohort with more reduced eGFR than that reported in the PROTECT trial, reinforcing the clinical relevance of the observed response [15, 16]. Kidney function remained stable during the 12-month follow-up despite the marked reduction in proteinuria. This finding contrasts with the significant decline in eGFR observed during the year preceding sparsentan initiation and is reassuring in a real-world population with moderately reduced baseline kidney function. However, given the relatively short follow-up and limited sample size, these observations should be interpreted cautiously and do not allow definitive conclusions regarding the long-term nephroprotective effect of sparsentan.

Real-world evidence on sparsentan remains limited. The series reported by Schanz *et al*. demonstrated a marked early reduction in proteinuria, with a decrease of ∼60% at around 14 weeks, in a cohort also treated with SGLT2i [17]. Preliminary results from the phase 2 SPARTACUS trial, presented as an abstract at Kidney Week 2025, also suggest that the addition of sparsentan to stable SGLT2 inhibitor therapy provides further reductions in proteinuria while maintaining an acceptable safety profile. Our findings are consistent with these data and extend them by showing that the antiproteinuric effect is maintained beyond the early follow-up period. The near-universal use of SGLT2i in our cohort further supports the relevance of these findings in the context of current clinical practice, where these agents have become part of standard care in IgAN [8, 9].

The distinction between responders and nonresponders provides additional clinical insight. In our cohort, >50% of patients achieved a reduction in proteinuria >50% at 3 months. Although this threshold does not necessarily represent disease remission, it reflects a clinically meaningful antiproteinuric response. Responders presented with hematuria at the time of sparsentan initiation more frequently than nonresponders. Although the exploratory multivariable analysis did not identify factors independently associated with treatment response, baseline hematuria was more frequent among responders and tended to disappear by month 3. This finding should be interpreted cautiously but may reflect a subgroup of patients with more active disease who retain a greater capacity for improvement. While purely speculative, this observation could be consistent with a potential anti-inflammatory effect of sparsentan, an aspect that warrants further investigation. During the follow up, less patients presented hematuria than at the time of sparsentan initiation. Given the multicenter retrospective design, its assessment was not standardized across centers, which may limit the interpretation of this finding. In addition, patients who achieved response tended to exhibit a greater reduction in blood pressure early after treatment initiation. This observation, although exploratory, is in line with findings from long-term observational data reported by Heerspink *et al*., where patients achieving complete remission (proteinuria <300 mg/day) over extended follow-up in PROTECT trial more frequently presented with lower blood pressure levels [19]. While the timescale and endpoints differ, both observations point toward a consistent signal suggesting that patients who experience a more pronounced hemodynamic response early on may be those more likely to achieve a favorable antiproteinuric profile, contributing to the observed variability in response.

More recently, longer-term real-world data from Schanz *et al*. showed a sustained antiproteinuric effect of sparsentan in patients receiving concomitant SGLT2 inhibition, with a 61% reduction in proteinuria at 12 months, slightly greater than that observed in our cohort. Kidney function also remained relatively stable over follow-up. These findings are consistent with our results and provide additional real-world evidence supporting the sustained effectiveness of sparsentan when added to contemporary supportive care [20].

Although the antiproteinuric effect of sparsentan was substantial, complete remission remained uncommon. At 12 months, only 21.7% of patients achieved proteinuria <0.5 g/24 h and 13.0% reached proteinuria <0.3 g/24 h, meaning that nearly 80% of patients still had residual proteinuria above 0.5 g/24 h despite treatment with sparsentan on top of SGLT2 inhibitors. While these results compare favorably with those reported in previous studies and represent a clinically meaningful reduction in proteinuria, they also highlight that a considerable proportion of patients remain at risk of disease progression. These findings emphasize the need for additional therapeutic strategies and further research to optimize disease control in patients with IgAN.

The consistency of response across sexes is also noteworthy. Despite women presenting with a higher prevalence of hematuria and lower systolic blood pressure at baseline, with these differences persisting at 3 months, no differences were observed between men and women in terms of proteinuria reduction or response rates. The relative reduction in systolic blood pressure was also similar between both groups, suggesting a relatively homogeneous treatment effect without an obvious modifying role of sex.

From a safety perspective, sparsentan was well tolerated, with no clinically relevant hyperkalemia and only mild, reversible episodes of AKI, findings that are consistent with previously reported data [15, 18, 19].

The limitations of this study include its retrospective design, the relatively small sample size, and the progressive reduction in the number of patients with longer follow-up. In addition, although 54 patients were initially considered, only 41 had sufficiently complete data to be included in the analysis. The assessment of hematuria should also be interpreted with caution, as it was not uniformly quantified across centers, reflecting the inherent variability of a multicenter real-world study. The absence of a control group also limits the ability to distinguish the specific contribution of sparsentan from the potential antiproteinuric effect of concomitant background therapies, particularly SGLT2 inhibitors. However, background treatment at the time of sparsentan initiation was relatively homogeneous across the cohort, with nearly all patients receiving SGLT2 inhibitors. The multicenter nature of the cohort and the inclusion of patients treated under routine clinical conditions strengthen the clinical applicability and external validity of the findings.

Taken together, these findings indicate that the antiproteinuric effect of sparsentan is not only reproducible outside the clinical trial setting but also sustained over time in patients treated according to current standards of care. In a real-world population characterized by prior optimization of therapy and widespread use of SGLT2 inhibitors, the magnitude and persistence of proteinuria reduction, together with stable kidney function, support the role of sparsentan as a meaningful therapeutic option in IgAN.

## Data Availability

The datasets generated and/or analyzed during the current study are not publicly available due to the presence of identifiable patient information. However, anonymized data can be made available from the corresponding author upon reasonable request, in compliance with institutional and regulatory requirements.
